# Perceptual organization and task demands jointly shape auditory working memory capacity

**DOI:** 10.1121/10.0025392

**Published:** 2024-03-25

**Authors:** Abigail L. Noyce, Leonard Varghese, Samuel R. Mathias, Barbara G. Shinn-Cunningham

**Affiliations:** 1 Neuroscience Institute, Carnegie Mellon University, Pittsburgh, Pennsylvania 15213, USA; 2 Department of Biomedical Engineering, Boston University, Boston, Massachusetts 02215, USA; 3 Department of Psychiatry, Boston Children's Hospital, Harvard Medical School, Boston, Massachusetts 02115, USA anoyce@andrew.cmu.edu, lvarg128@outlook.com, samuel.mathias@childrens.harvard.edu, bgsc@andrew.cmu.edu

## Abstract

Listeners performed two different tasks in which they remembered short sequences comprising either complex tones (generally heard as one melody) or everyday sounds (generally heard as separate objects). In one, listeners judged whether a probe item had been present in the preceding sequence. In the other, they judged whether a second sequence of the same items was identical in order to the preceding sequence. Performance on the first task was higher for everyday sounds; performance on the second was higher for complex tones. Perceptual organization strongly shapes listeners' memory for sounds, with implications for real-world communication.

## 1. Introduction

Parsing acoustic input into “objects”—that is, separating a mixture of sound into estimates of the content of discrete physical sources—underlies our ability to analyze and make sense of what we hear (Bregman, 1994; Griffiths and Warren, 2004; Shinn-Cunningham, 2008; Shinn-Cunningham *et al.*, 2017). This process depends not only on the moment-by-moment properties of the auditory input, but also on temporal context (Cowan, 1984; Krumhansl and Iverson, 1992; Massaro, 1972; Shamma *et al.*, 2011; Tervaniemi *et al.*, 1994; Tiitinen *et al.*, 1994; Warrier and Zatorre, 2002). A sequence of individual events may be organized into one object that extends through time, as in a melody made up of a series of individual notes, or, at the other extreme, into a series of separate objects.

We hypothesize that such perceptual organization affects not only attention, but also short-term memory. That is, when people are asked to remember some auditory input, the representation in which they store that information and their subsequent ability to retrieve it will depend on its perceived structure. When a sequence of sounds is heard as a single, temporally extended object, people are quite good at discriminating changes in the serial order of elements within the sequence (Dowling and Fujitani, 1971; Fitzgibbons and Gordon-Salant, 1998); however, they may find it difficult to access the individual elements (Billig and Carlyon, 2016; Cusack *et al.*, 2004). When sequences are composed of dissimilar sounds, listeners need to re-orient attention to each subsequent sequence element (Shamma *et al.*, 2011; Shinn-Cunningham *et al.*, 2017) and have a difficult time recalling their relative order (Warren *et al.*, 1969; Warren and Obusek, 1972).

Here, we demonstrate this relationship between perceptual organization and aspects of recall by measuring short-term memory for short sequences of auditory items. We developed two sets of stimuli and two tasks that would emphasize different aspects of memory representation. One task asked subjects to listen to a short sequence, hold it in memory, and then report whether or not a specific probe item had been present in the preceding sequence (loosely after Sternberg, 1966), a task that would be difficult if the sequence was remembered as a single stream, but relatively easy if the elements were encoded as separate objects, each of which could be independently accessed. The second task asked subjects to listen to two short sequences comprising the same elements and determine whether or not all items occurred in the same sequential order, a task that would be relatively difficult if the elements were remembered as independent objects but relatively easy if the sequence was encoded as a single stream whose identity depended on the relative order of its elements.

One set of stimuli were broadband tones differing only in pitch, likely to be heard and encoded as a single auditory object. The other set of stimuli were spectrotemporally dissimilar “everyday sounds,” which were likely to be heard and encoded as discrete objects.

## 2. Methods

### 2.1 Subjects

Younger adults (*N* = 14) were recruited from the Boston University community. Each subject provided written informed consent and was paid for their time. All subjects had normal hearing, defined as audiometric thresholds of less than 20 dB hearing level in each ear across test frequencies from 250 to 8000 Hz. Although some subjects reported having musical training, none were professional musicians, and none reported having absolute pitch.

### 2.2 Stimulus elements

All stimulus elements were manipulated in matlab (The MathWorks, Inc., Natick, MA) using a sampling rate of 48.828 kHz. We used two distinct sets of 16 elements each.

**Fig. 1.**
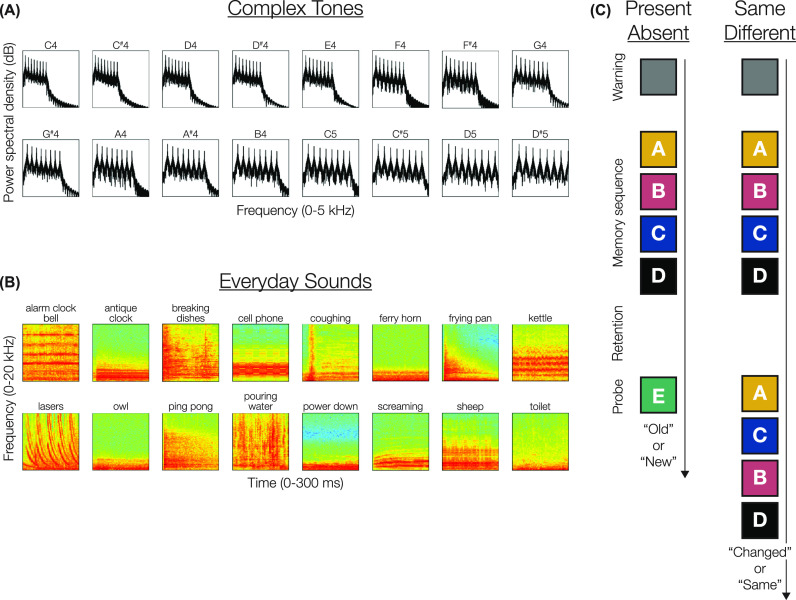
Stimuli and task schematics. (A) One stimuli set comprised 16 300 ms complex tones (power spectra illustrated here), with fundamental frequencies between C4 (∼262 Hz) and D#5 (∼623 Hz) in semitone steps. (B) The other stimuli set comprised 16 300 ms snippets of “everyday sounds” (Cohen *et al.*, 2009). (C) In each task, a memory sequence of 4, 6, 8, or 10 tones was presented, followed by a retention interval. In the present/absent task (left), a single probe item was then played, and subjects judged whether it had been part of the memory sequence. In the same/different task (right), a second sequence of the same items was played, and subjects judged whether it was identical or if the serial order of items had changed.

The first set was made up of complex tones with fundamental frequencies between C4 (∼262 Hz) and D#5 (∼623 Hz) in semitone steps, as on the Western musical scale [Fig. 1(A)]. To create sounds with similar timbre and with a relatively broadband spectrum, we created 300 ms periodic waveforms comprising the first eight harmonics of a sawtooth waveform (i.e., summing eight sinusoids whose frequencies are multiples of the fundamental, with the harmonics weighted by 1, –1/2, 1/3, –1/4, …, 1/8, respectively). Note that semitone steps are comfortably larger than typical just-noticeable differences for human pitch perception (Houtsma and Smurzynski, 1990; Moore, 1973), and this range of frequencies falls within the high-sensitivity range of human hearing (Quam *et al.*, 2012).

The second set comprised 300-ms-long 16 snippets of “everyday sounds” [Fig. 1(B)] selected from the corpus presented by Cohen *et al.* (2009). These sounds were selected to vary in both their spectrotemporal structure at a variety of scales and in their semantic associations. At this duration, these sounds were distinct from each other in their quality; however, it was difficult to process meaningful semantic labels for each sound given their brief durations and the relatively rapid presentation rate (see below).

All stimulus elements were windowed with 5-ms-long raised cosine-squared ramps at both their onsets and offsets, after which the levels of the individual elements were equalized to have the same root mean squared energy before being combined into memory sequences and probes (as described below). Stimuli were presented diotically at a comfortable, listener-adjusted level via a Tucker-Davis Technologies (Alachua, FL) RP2 processor and Etymotic (Elk Grove, IL) ER-1 insert earphones.

### 2.3 Experimental tasks and design

Each participant completed two different experimental tasks (present/absent and same/different; Fig. 1(C)). At the start of each trial in both tasks, participants first heard a 1-s-long burst of white noise as a warning that a new trial was starting, followed by a 500-ms-long pause. They were then presented with a short to-be-remembered sequence of either the complex tones or the everyday sounds. The structure of the memory sequences was identical across tasks and across stimuli sets (complex tones or everyday sounds).

Each memory sequence consisted of 4, 6, 8, or 10 elements, which were randomly sampled without replacement from the 16 elements of that trial's stimuli set. All experiments were blocked by both sequence length and stimulus set (see below). Within each memory sequence, the selected elements were separated by a 100 ms silent interstimulus interval. In both tasks, subjects had to maintain this sequence in memory over a 2100 ms retention interval, after which they were presented with a probe, which differed for the two tasks. After the probe, a response prompt (also differing for the two tasks) appeared on the display, at which time subjects responded via the keyboard with “Y” for “yes” and “N” for “no.” No limit was imposed on response time, and response times were not collected or analyzed. After a response was entered, feedback was presented on the computer screen informing the subject as to whether they were correct or incorrect on that trial. An auditory masker (1000 ms of broadband noise) and a brief (500 ms) silent intertrial interval occurred before the onset of the next trial.

#### 2.3.1 Present/absent task

In the present/absent task, the probe was a single element from that trial's stimuli set. The probe was equally likely to be a member of the memory sequence or one of the remaining items. When drawing a probe from the memory sequence, its serial position was selected at random. The response prompt in this task read, “Was the probe in the original sequence?”

#### 2.3.2 Same/different task

In the same/different task, the probe was equiprobably either an exact duplicate of the memory sequence or identical, except with two adjacent elements switched in order. When two adjacent items were to be switched, their serial position in the memory sequence was selected randomly. The response prompt in this task read, “Were the two sequences identical?”

#### 2.3.3 Experimental design and procedures

Trials were organized in blocks of ten trials each that shared the same task (present/absent or same/different), stimulus set (complex tones or natural sounds), and sequence length (4, 6, 8, or 10) for a total of 16 different block types. Each subject participated in data collection over multiple days (sessions). Within each session, subjects typically performed one set of each block type in a randomly generated (different for each subject) order and then a second set of each block type in a different random order, leading to a total of 32 blocks per session (20 trials of each combination of task, stimuli set, and sequence length). Eleven participants completed six sessions (120 trials for each combination of task, stimuli set, and sequence length; 1920 trials total) and three completed five sessions (100 trials for each condition; 1600 trials total). Electroencephalography was collected from each participant; those results are not presented here.

### 2.4 Analyses

Sensitivity (*d′*) was computed for each subject, stimulus set, sequence length, and task. Any time a condition's hit or false alarm rate was exactly 1.0 or 0.0, the count of correct trials was adjusted down or up by 0.5 trials, respectively, prior to computing *d′*.

We also conducted *post hoc* analyses of accuracy across serial positions. Proportion correct was calculated for each serial position of the probe item (for the present/absent task) or the swapped items (for the same/different task). We defined the “middle” of each sequence to exclude the first and last item (in the present/absent task) or swaps involving those items (in the same/different task). A linear mixed-effects model was fit to the accuracy across middle serial positions for each task and sequence length (6, 8, and 10 items) separately. This model had serial position, stimulus set, and the interaction between them as predictors. In order to understand whether the slopes of these models were significantly different between the two tasks, we fit a third model for each sequence length (6, 8, and 10 items), which had serial position, stimulus set, and task, as well as all two- and three-way interactions as predictor variables.

When we conducted multiple statistical tests (e.g., a family of *t* tests), the Holm-Bonferroni method was used to correct for multiple comparisons.

## 3. Results

### 3.1 Overall performance

**Fig. 2.**
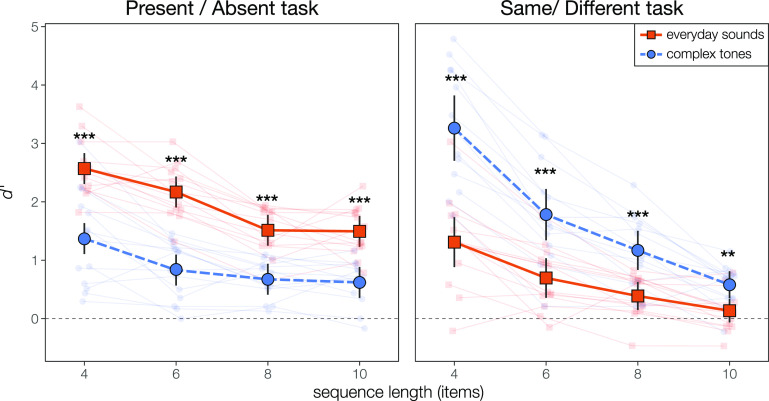
Performance (*d′*) by task, stimuli set, and sequence length. In the present/absent task (left), listeners were significantly better when the stimuli were everyday sounds than when they were complex tones. In the same/different task (right), listeners were significantly better when the stimuli were complex tones than when they were everyday sounds. Both effects held at every sequence length. ^*^p < 0.05; ^**^p < 0.01; ^***^p < 0.001; Holm-Bonferroni corrected for 8 comparisons.

There was a clear double dissociation between task and stimuli type. In the present/absent task, subjects were more sensitive to a single probe item for everyday sounds than for complex tones (Fig. 2, left). However, subjects were more sensitive to a switch on the order of sound elements in the same/different task when the sequence elements were complex tones than when they were everyday sounds (Fig. 2, right). Both effects were statistically significant at every sequence length (paired *t* tests, Holm-Bonferroni corrected for 8 comparisons, all *p* < 0.002).

Within each task, we fit a linear mixed-effects model with fixed effects of sequence length and sound type and a random intercept per subject. Permutation testing (Holm-Bonferroni corrected for six comparisons) confirmed that both tasks exhibited a significant linear effect of sequence length [Present/Absent: F(1,95) = 103.08, p < 0.001; Same/Different: F(1,95) =  257.725, p < 0.001], a significant effect of sound type [Present/Absent: F(1,95) = 235.4118, p < 0.001; Same/Different: F(1,95) = 150.03, p < 0.001], and a significant interaction between them [Present/Absent: F(1,95) = 5.64, p = 0.020; Same/Different: F(1,95) = 938.48, p < 0.001].

### 3.2 Serial position effects

**Fig. 3.**
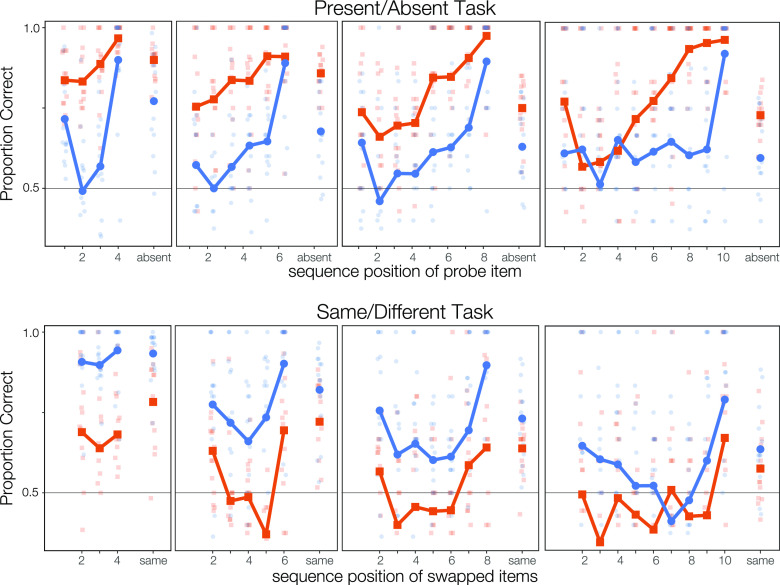
Accuracy on each task (top: Present/Absent; bottom: Same/Different) for each stimulus type (orange: everyday sounds; blue: complex tones) for each sequence length and serial position. Dots show individual subjects' performance; the range is very large.

In order to better understand how performance for these two sets of stimuli differed, we plotted the proportion correct at each serial position for each task, sound type, and sequence length (Fig. 3). For both types of stimuli in both tasks, we saw evidence of well-established primacy and recency effects on recall (Atkinson and Shiffrin, 1968; Murdock, 1962). However, beyond that, the serial position curves for each task suggest that participants were able to access and recall stored information differently for the two types of stimuli. Particularly for everyday sounds, performance in the present/absent task (Fig. 3, top panels) tended to increase with serial position, with recall accuracy for the final and penultimate items very high; in contrast, in the same/different task (Fig. 3, bottom panels), performance was very nearly flat with serial position.

Subjects were generally able to perform the present/absent task for everyday sounds at levels above chance (Fig. 3, top, orange points). They were always significantly above chance accuracy at rejecting lure probes (rightmost points in each plot; p values are Holm-Bonferroni corrected for 120 comparisons). When probed with targets, subjects were significantly above chance when the probe item had appeared within five or six positions from the end, regardless of sequence length.

These results contrast with those for the present/absent task with complex tones (Fig. 3, top, blue points). Subjects were significantly above chance accuracy at rejecting lures only in 4- and 6-item sequences. Further, subjects were significantly above chance at identifying targets only when they either occurred in the last sequence position (regardless of sequence length) or were the first item in the shortest, 4-item sequences.

Accuracy was somewhat lower on the same/different task (Fig. 3, bottom panels) than on the present/absent task. Subjects were significantly above chance at identifying changes in sequence order for complex tones at all serial positions in 4-item sequences; at the first and last position in 6-item sequences; but only when the final two items were switched for the longer sequences (Fig. 3, bottom, blue points; p values are Holm-Bonferroni corrected for 120 comparisons). Subjects correctly identified “same” sequences of complex tones at levels better than chance for all sequence lengths except ten items. For everyday sounds, however, subjects were much worse. They were unable to identify sequence changes at any sequence length or serial position at above-chance levels (with correction for multiple comparisons). However, they were able to correctly identify “same” sequences for lengths 4, 6, and 8 (Fig. 3, bottom, orange points).

For sequences of lengths 6, 8, and 10, we isolated the middle sequence elements and, for each task at each sequence length, fit a linear mixed-effect model with fixed effects of serial position, stimulus set, and the interaction between those factors, and with random intercepts per subject. This allowed us to estimate the slope term associated with serial position, for each task and sequence length. The fitted slopes are given in Table 1.

**Table 1.** Estimated effect of serial position (measured as the slope of performance by position) for the middle items of each task and sequence length. Slope estimates are in units of increased hit rate per increase in serial position. Slopes are collapsed across stimulus set in all but one condition because they do not significantly differ. ^*^p < 0.001; ^**^p < 0.10; Holm-Bonferroni corrected for 7 comparisons.

Task	Sequence length	Slope estimate (standard error)
Present/absent	6	0.049 (0.028)
	8	0.062 (0.015)^*^
	10 (everyday sounds)	0.062 (0.012)^*^
	10 (complex tones)	0.013 (0.014)
Same/different	6	−0.045 (0.033)
	8	0.391 (0.017)^**^
	10	0.008 (0.011)

On the present/absent task, these middle elements show a consistently positive slope, with each increment in position accounting for 5 or 6 percentage points of increased accuracy. This slope is significantly above zero in 8-item sequences, regardless of stimuli set, and in the 10-item sequences when listeners recognize everyday sounds. (The only model with a significant interaction between serial position and stimuli set was the 10-item sequences on this task; they are reported separately here, and the tones notably lack this effect.) In contrast, the middle elements of the same/different task do not exhibit any consistent pattern. The slope is slightly negative for 6-item sequences, marginally positive for 8-item sequences, and almost flat for 10-item sequences.

We then combined the two tasks into a larger model, with task (and its interactions) as explicit predictors. In order to test whether the effect of serial position differed between tasks, we assessed whether either the interaction between task and serial position, or the three-way interaction between task, stimuli set, and serial position, were significant. For 10-item sequences, there was a significant task by serial position interaction (p = 0.0009); for 6-item sequences, the interaction was marginal (p = 0.059). No other interactions of task and serial position were significant (all p > 0.339).

## 4. Discussion

We created two types of short auditory sequences whose elements were either very similar (complex tones) or dissimilar (everyday sounds). These short sequences were then used in two working memory tasks: one in which listeners had to determine whether an isolated stimulus element had been a member of the sequence (present/absent) and one in which listeners had to identify whether the order of stimuli in the sequence had changed (same/different). We found that listeners were better able to recognize individual items when the sequence was composed of everyday sounds, but better able to recognize changes in order when the sequence was composed of complex tones, even though the structure and timing of the presentation of the sequences were identical across stimulus types. These results suggest that perceptual organization fundamentally shapes how information is encoded into, and can be accessed from, working memory.

### 4.1 Element similarity and memory effects

Listeners were able to perform the present/absent task reasonably well for the everyday sounds (mean *d′* near or above 2 for all sequence lengths), but struggled with the same/different task (mean *d′* below 1 for sequences of six or more items). Because the everyday sounds are so different from one another, they are likely perceived as a series of individual objects, each of which is stored as a discrete event in memory, allowing listeners to directly recall individual elements. If the everyday sounds in the memory sequence are stored as independent events, their temporal context is likely a separate, associated memory trace (Howard and Kahana, 2002; Manns *et al.*, 2007). Detecting changes in the temporal order in the same/different task thus requires comparisons between separate memory representations, leading to poor performance, especially for items early in the sequence (Howard *et al.*, 2015; Singh *et al.*, 2018). These challenges in accessing temporal order may be exacerbated at the encoding stage by the cost of switching attention between individual auditory objects (Billig and Carlyon, 2016; Bregman and Campbell, 1971; Cusack *et al.*, 2004; Hughes *et al.*, 2011; Lim *et al.*, 2019).

Conversely, listeners were quite successful at discriminating changes in temporal order for the complex tones (*d′* > 3 for short sequences, and significantly higher than everyday sounds at all sequence lengths), but generally had difficulty performing the same task with complex tones (*d′* near or below 1 for all sequence lengths). This dissociation cannot be explained by a simple inability to recall tone information (Akiva-Kabiri *et al.*, 2009; Li *et al.*, 2013) or to discriminate among the pitches (which are comfortably above the just noticeable difference for human pitch perception). One likely explanation is that listeners perceive the complete sequence as a single object, and that object percept is stored in short-term memory as a single unit, accessible as a sequence (Mathias and von Kriegstein, 2014; Zimmermann *et al.*, 2016). The order of pitches thus helps define the object identity, stored intrinsically as part of the memory representation (Bizley and Cohen, 2013; Griffiths and Warren, 2004). Any change in sequence order results in a change to the object identity, making it relatively easy to detect. However, this unitary memory representation may preclude direct access to the representation of individual tones (Billig and Carlyon, 2016; Cusack *et al.*, 2004; Jones *et al.*, 1997).

Subjective reports from our subjects are consistent with these ideas. In particular, for complex tone stimuli, subjects felt that, when trying to do the present/absent task, they were forced to “play through” the entire stored memory sequence, in order, and compare each individual element, in turn, with the probe tone; moreover, they reported that the internal playback of the memory sequence interfered with their ability to maintain the probe tone that they had to compare to each item in the sequence being recalled.

### 4.2 Serial position effects

We observed a marked primacy effect in many conditions. Superficially, this result appears to be at odds with the finding that perceptual continuity aids in focusing attention and processing information in a scene, resulting in recency, but not primacy, benefits (e.g., Best *et al.*, 2008; Bressler *et al.*, 2014). However, in those studies, listeners had to select and follow one target speech source from among multiple competing speech streams. Our task was not designed to require attentional selection, as only one stream was presented, making it easy to immediately encode each heard item into short-term memory.

The other striking finding is the generally monotonic increase across serial positions for the present/absent task, especially for everyday sounds. For these stimuli, results for this task illustrate a classic recency effect, wherein the time and the number of intervening items between the target's first appearing in the memory sequence and its reappearance as the probe lead to degraded memory. Performance for the same/different task is very different—largely flat across serial positions other than first and last—illustrating its dependence on relationships among items rather than the item identities themselves.

## 5. Conclusion

When asking listeners to remember auditory sequences, we observed a strong interaction between the type of memory task (recognizing a single sequence element in isolation vs recognizing changes in the temporal order of the sequence) and the type of stimuli (complex tones vs snippets of everyday sounds). Listeners were reliably better at recognizing a single everyday sound than a single tone, but better at recognizing the order of tones than the order of everyday sounds. These results provide further evidence that perceptual organization of sound sequences into auditory objects strongly shapes how they are encoded into and retrieved from working memory. However, further work is required to fully understand which features of an acoustic sequence most strongly drive its storage into memory as a single item rather than a series of discrete elements.

## Data Availability

The data that support the findings of this study are available from the corresponding author upon reasonable request.
